# Spontaneous Primary Intraventricular Hemorrhage: Clinical Features and Early Outcome

**DOI:** 10.5402/2012/498303

**Published:** 2012-08-26

**Authors:** Adrià Arboix, Luis García-Eroles, Adela Vicens, Montserrat Oliveres, Joan Massons

**Affiliations:** ^1^Unidad de Enfermedades Cerebrovasculares, Departamento de Neurología, Capio-Hospital Universitari del Sagrat Cor, Universitat de Barcelona, Catalonia, 08029 Barcelona, Spain; ^2^CIBER de Enfermedades Respiratorias (CB06/06), Instituto de Salud Carlos III, Madrid, Spain; ^3^Direcció d'Organització i Sistemes d'Informació, Gerència Territorial Metropolitana Nord, Institut Català de la Salut (ICS), Badalona, Catalonia, Barcelona, Spain

## Abstract

*Purpose*. Primary hemorrhage in the ventricular system without a recognizable parenchymal component is very rare. This single-center retrospective study aimed to further characterize the clinical characteristics and early outcome of this stroke subtype. *Methods*. All patients with primary intraventricular hemorrhage included in a prospective hospital-based stroke registry over a 19-year period were assessed. A standardized protocol with 161 items, including demographics, risk factors, clinical data, neuroimaging findings, and outcome, was used for data collection. A comparison was made between the groups of primary intraventricular hemorrhage and subcortical intracerebral hemorrhage. Predictors of primary intraventricular hemorrhage were identified by logistic regression analysis. *Results*. There were 12 patients with primary intraventricular hemorrhage (0.31% of all cases of stroke included in the database) and 133 in the cohort of subcortical hemorrhage. Very old age (≥85 years) (odds ratio (OR) 9.89), atrial fibrillation (OR 8.92), headache (OR 6.89), and altered consciousness (OR 4.36) were independent predictors of intraventricular hemorrhage. The overall in-hospital mortality rate was 41.7% (5/12) but increased to 60% (3/5) in patients aged 85 years or older. *Conclusion*. Although primary intraventricular hemorrhage is uncommon, it is a severe clinical condition with a high early mortality. The prognosis is particularly poor in very old patients.

## 1. Introduction

Intraventricular hemorrhage is a common complication of parenchymal intracerebral hemorrhage and subarachnoid hemorrhage [1, 2]. However, spontaneous primary intraventricular hemorrhage without a recognizable parenchymal component in noncontrast-enhanced computed tomography (CT) or magnetic resonance imaging (MRI) studies is extremely rare [3]. Limited data on the clinical characteristics and outcome of patients with spontaneous primary intraventricular hemorrhage is partially due to rarity of the condition and the fact most studies of primary intracerebral hemorrhage are focused on the global assessment of patients with hemorrhagic stroke [1, 2]. It has been shown that the clinical spectrum, prognosis, and early mortality in patients with primary intracerebral hemorrhage are reasonably dependent on the site of bleeding [4]. On the other hand, hemorrhages in the thalamus, basal ganglia, and internal capsule are a group of well-known supratentorial cerebral hemorrhages of subcortical topography, with a clinical profile that is clearly different from those of other topographies, including lobar, cerebellar, brainstem, and intraventricular hemorrhages [2]. 

 This single-center retrospective study aimed to further characterize the clinical profile, risk factors, prognosis, and early outcome of patients with primary intraventricular hemorrhage. A secondary objective was to assess clinical differences between patients with primary intraventricular hemorrhage and patients with subcortical hemorrhage. Data for this study were collected from a prospective hospital-based stroke registry in Barcelona, Spain.

## 2. Materials and Methods

Patients for this study were collected from Registro de Ictus del Hospital del Sagrat Cor de Barcelona, which is an ongoing prospective hospital-based stroke registry, in which data from stroke patients admitted consecutively to the Department of Neurology of Sagrat Cor hospital (an acute-care 350-bed teaching hospital in the city of Barcelona, Spain) are entered following a standardized protocol. Details of the database have been previously described [4, 5]. The Cerebrovascular Study Group of the Spanish Neurological Society [6] was used for the classification of subtypes of stroke, which included transient ischemic attack, cerebral infarction (thrombotic, cardioembolic, lacunar, unusual etiology, and unknown cause), intracerebral hemorrhage, subarachnoid hemorrhage, spontaneous subdural hematoma, and spontaneous epidural hematoma. This classification is similar to that of the National Institute of Neurological Disorders and Stroke Classification [7]. Definitions of cerebrovascular risk factors have been used by our group in previous studies [4, 5].

**Figure 1:**
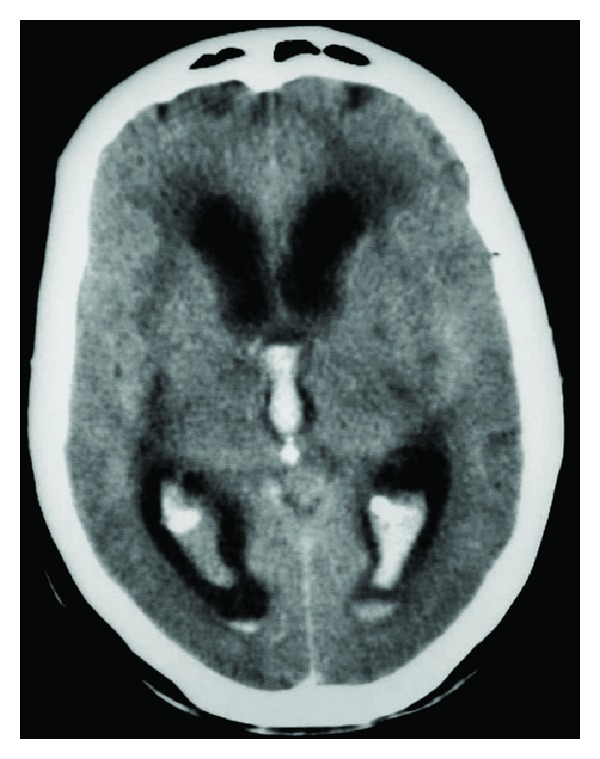
Primary intraventricular hemorrhage in the brain CT scan.

For the purpose of this study, data from patients with primary intraventricular hemorrhage attended over a 19-year period were collected. Patients were diagnosed with primary intraventricular hemorrhage when brain CT and/or MRI studies revealed blood restricted to the ventricular system (Figure 1). Patients with intraparenchymatous hemorrhage were excluded even if the hemorrhage was small or very close to the ventricular system or subarachnoid blood, as were patients with history of head trauma. For comparative purposes, data from patients with subcortical hemorrhage (basal ganglia and internal capsule) were also collected. Prior to conducting the study, approval was obtained from the Ethical Committee of Clinical Research of the hospital.

 All patients were admitted to the hospital within 48 hours of onset of symptoms. On admission, demographic characteristics, salient features of clinical, neurological examination and results of laboratory tests (blood cell count, biochemical profile, serum electrolytes, urinalysis), chest radiography, twelve-lead electrocardiography, and brain CT and/or MRI were recorded. Other investigations, including angio-MRI, arterial digital subtraction angiography, echocardiography, and lumbar puncture were obtained at discretion of the attending physician. The degree of clinical disability at discharge from the hospital was evaluated according to modified Rankin scale (mRS) [8] and causes of death according to the criteria of Silver et al. [9].

### 2.1. Statistical Analysis

Demographic characteristics, risk factors, clinical events, and outcome of patients with primary intraventricular hemorrhage and those with subcortical hemorrhage were compared using the Student's *t*-test or the Mann-Whitney *U* test for continuous variables and the chi-square (*χ* ^2^) test (with Yate's correction when necessary) for categorical variables. Variables were subjected to multivariate analysis with a logistic regression procedure and forward stepwise selection if *P* < 0.10 after univariate testing. The effect of variables on the presence of primary intraventricular haemorrhage was studied in a multiple regression model based on demographic, vascular risk factors, and clinical and neuroimaging variables, in which primary intraventricular hemorrhage was the dependent variable. Statistical significance was set at *P* < 0.05.

## 3. Results

A total of 12 patients with primary intraventricular hemorrhage were collected. These cases accounted for 0.31% of all cases of stroke included in the database (*n* = 3808) and 3.3% of all cases of intracerebral hemorrhage (*n* = 407) included in the database. There were 5 men and 7 women, with a mean (standard deviation, SD) age of 78.9 (7.2) years. Five patients (41.7%) aged 85 years or more, and all of these patients were females. Hypertension (41.7%), vascular anomaly (16.7%), anticoagulation (16.7%), and hematological conditions (8.3%) were the main causes of primary intraventricular hemorrhage. The aetiology of HIV bleeding was not identified in 16.7% of patients. Risk factors, clinical characteristics, and outcome are shown in Table 1. One patient had surgical intervention (ventricular drainage). Median duration of hospital stay was 18.5 days. At the time of hospital discharge, 1 patient (8.3%) was symptom-free (mRS grade 0). Of the remaining 11 patients, 2 had moderate disability (mRS grade 3), 2 moderately severe disability (mRS grade 4), and 2 severe disability (mRS grade 5).

A total of 5 patients died, with an in-hospital mortality rate of 41.7%. Three of these patients aged 85 years or more (in-hospital mortality rate 60%). The median time from the onset of symptoms to death was 11 days (25th−75th percentile, 6–13.5 days). Causes of death were cerebral herniation in 3 patients, pneumonia in 1, sepsis in 1, and unknown cause in 1.

Patients with primary intraventricular hemorrhage as compared with patients with subcortical hemorrhage (*n* = 133) were older (78.9 (7.2) *versus* 72.2 (11.9) years, *P* = 0.51), showed a higher percentage of patients aged 85 years or older (41.7% *versus* 14.3%, *P* = 0.029), patients with valve heart disease (16.7% *versus* 2.3%, *P* = 0.055), atrial fibrillation (41.7% *versus* 12.0%, *P* = 0.016), headache at stroke onset (50% *versus* 24.8%, *P* = 0.066), altered consciousness (66.7% *versus* 29.3%, *P* = 0.012), and in-hospital mortality rate (41.7% *versus* 16.5%, *P* = 0.048).

 In the multivariate analysis, factors independently associated with primary intraventricular hemorrhage were 85 years old or more, atrial fibrillation, headache, and altered consciousness (Table 2).

## 4. Discussion

Data regarding the frequency of primary intraventricular hemorrhage in the different hospital-based stroke registries are scarce the present results show that primary intraventricular hemorrhage is a rare subgroup of hemorrhagic stroke that accounted for 0.31% of all cases of stroke and 3.3% of intracerebral hemorrhages. The prevalence of primary intraventricular hemorrhage in different clinical series of intracerebral hemorrhage varies widely from 2% in the series of Hameed et al. [10] to 7% in the series of Ara et al. [11]. In a subsample of 551 with hemorrhagic stroke reported by Flint et al. [12], primary intraventricular hemorrhage was diagnosed in 15 patients (2.7%).

 We found that patients with primary intraventricular hemorrhage and patients with subcortical haemorrhage presented different clinical profiles, with 85 years old or more, atrial fibrillation, headache at stroke onset, and altered consciousness being significantly more frequent in patients with primary intraventricular hemorrhage.

 A remarkable finding of our study is the advanced mean age of patients with primary intraventricular hemorrhage of 78.9 years, with 41.7% of patients aged 85 years or more as compared with the mean age of patients with subcortical hemorrhage (72.2 years) as well as the mean age of 60 years in patients with primary intraventricular hemorrhage reported by Martí-Fàbregas et al. [13] and of 56 years in the series of Hameed et al. [10]. Also, 85 years of age or older was the main independent factor related to primary intraventricular hemorrhage. This aspect has not been previously reported and may be related to the increasing incidence of stroke in the oldest old segment of the population [14–18]. Also, elderly stroke patients are particularly at risk of receiving suboptimal care and there is evidence that brain neuroimaging and other diagnostic tools are less frequently used in the very old patients with acute stroke [19–21]. The present study carried out in the context of a prospective acute stroke registry ensures that all patients independently of their age at presentation underwent the same protocolized work-up studies. It should be noted that we have reviewed our experience with primary intraventricular hemorrhage over 19 years and a limitation is that management practices changed over this time period so this could impact the results. Because of the rarity of primary intraventricular hemorrhage, results of the present study should be interpreted taking into account that only 12 patients were included in the study. This limitation also applies to results of the multivariable analysis.

Atrial fibrillation was significantly more frequent in patients with primary intraventricular hemorrhage than in patients with subcortical hemorrhage. The occurrence of atrial fibrillation increases with age and the attributable risk of stroke for atrial fibrillation increased significantly rising from 1.5% for patients in the 50–59 years old group to 23.5% for those aged 80–89 years [22]. Headache at the time of stroke onset is more frequent in hemorrhagic than in ischemic stroke [23] and was found to be also more common in primary intraventricular hemorrhage than in subcortical hemorrhage. Headache, probably secondary to acute intracranial hypertension, was experienced by 50% of patients in our study, which is lower than 78% reported in the series of Angelopoulos et al. [24]. Also, the state of alertness of the patient is a clinical feature that correlates with prognosis in hemorrhagic stroke and, in general, in acute stroke patients. Reduced alertness in intracerebral hemorrhage is due to either a generalized increase in intracranial pressure, or to compromise of both hemispheres, to the reticular activating system bilaterally in the brainstem tegmentum [1, 3].

 Primary intraventricular haemorrhage is a severe clinical condition with an in-hospital mortality rate of 41.7%. Only one patient (8.3%) was symptom-free at discharge from the hospital. In the series of Passero et al. [25], Angelopoulos et al. [24], and Verma et al. [26], the case fatality was 43%, 36%, and 33.3%, respectively. As shown in Table 3, early mortality rate usually ranges between 20% and 45%, although in a few series lower figures were reported [10, 12, 27]. In the study of Tembl et al. [27], the in-hospital mortality was 0%, which is similar to that observed by our group in hemorrhagic lacunar stroke [28]. We also found that very old patients with acute stroke showed a differential clinical profile, different frequency of stroke subtypes, and a poorer outcome compared with stroke patients who were younger than 85 years of age [29]. Death increased with age and in agreement with the study of Daverat el al. [30], age was the most important predictor of death and functional outcome after spontaneous intracerebral hemorrhage. Massive primary intraventricular hemorrhage that produces fourth ventricular distension and periventricular cerebral compression is an especially ominous sign associated with rapid and advances neurological deterioration and with early death.

We found that all 5 patients aged ≥85 years were females. This high percentage of female patients among the oldest old is consistent with other studies [18, 19]. On the other hand, very old patients showed a worse prognosis as none of the patients was symptom-free at hospital discharge and the in-hospital mortality rate in this subgroup was 60% as compared with 28.6% in the remaining patients.

 Decompressive surgery in the management of primary intraventricular hemorrhage is not generally recommended. Studies in animal models studies have shown that clot removal can improve the acute and long-term consequences of intraventricular extension from intracerebral hemorrhage by using minimally invasive techniques coupled to recombinant tissue plasminogen activator-mediated clot lysis [31]. Also it has been shown that thrombolytic drugs administered intraventricularly through an external ventricular drain to lyse an intraventricular clot are safe and may reduce morbidity and mortality [32]. These findings will probably determine a change in the management of patients with acute intracerebral hemorrhage, in which ultraearly clot removal therapy, similar to the efficacy of early thrombolytic therapy in cerebral infarction, followed by careful monitorization in specialized stroke units could be of paramount importance in the care of patients with intracerebral hemorrhage [31–36].

 In summary, 3.3% of patients with acute hemorrhagic stroke had a primary intraventricular hemorrhage. Patients with primary intraventricular hemorrhage showed a differential clinical profile than patients with classical subcortical hemorrhages, mainly the higher frequency of presentation of very old age, a demographic feature that has not been previously observed. Primary intraventricular haemorrhage is a severe clinical condition with an in-hospital mortality rate of 41.7%. 

## Figures and Tables

**Table 1:** Data of 12 patients with spontaneous primary intraventricular hemorrhage.

Variable	No. patients (%)
Male sex	5 (41.7)
Patients aged ≥ 85 years	5 (41.7)
Vascular risk factors	
Hypertension	8 (66.7)
Diabetes mellitus	0
Hyperlipidemia	0
Valve heart disease	2 (16.7)
Atrial fibrillation	5 (41.7)
Ischemic heart disease	1 (8.3)
Heart failure	0
Peripheral vascular disease	1 (8.3)
Chronic liver disease	0
Smoking (>20 cigarettes/day)	1 (8.3)
Alcohol use (>80 g/day)	0
Oral anticoagulation	2 (16.7)
Clinical findings	
Sudden onset (minutes)*	8 (66.7)
Headache	6 (50)
Vertiginous symptoms	1 (8.3)
Nausea, vomiting	2 (16.7)
Altered consciousness	8 (66.7)
Motor deficit	8 (66.7)
Sensory deficit	4 (33.3)
Speech disturbance (aphasia, dysarthria)	2 (16.7)
Ataxia	0
Cranial nerves involvement	0
Lacunar syndrome	0
Outcome	
Neurological complications	4 (33.3)
Cardiological complications	1 (8.3)
Respiratory complications	1 (8.3)
Infectious complications	1 (8.3)
Symptom-free at hospital discharge	1 (8.3)
In-hospital mortality	5 (41.7)
Length of hospital stay, mean (25th–75th percentile)	18.5 (2.5–30)

*Sudden onset occurred in 1 (8.3%) patient aged <85 years and in 7 (100%) aged ≥85 years (*P* = 0.010).

**Table 2:** Results of multivariate analysis: predictors of primary intraventricular haemorrhage.

Variable	*β*	SE (*β*)	Odds ratio (95% confidence interval)	*P* value
Age 85 years or more	2.292	0.843	9.89 (1.89 to 51.63)	0.007
Atrial fibrillation	2.188	0.770	8.92 (1.97 to 4.34)	0.004
Headache	1.931	0.802	6.89 (1.43 to 33.17)	0.016
Altered consciousness	1.473	0.712	4.36 (1.08 to 17.59)	0.038

Model based on demographic variables, vascular risk factors, and clinical features (*β* = −5.042, SE (*β*) = 0.912, goodness-of-fit test *χ* ^2^ = 1.824, *df* = 4, *P* = 0.768). Area under the ROC curve = 0.894, sensitivity 83%, specificity 83%, positive predictive value 31%, negative predictive value 98%, diagnostic accuracy 83%.

**Table 3:** Primary intraventricular haemorrhage. Main series reported in the literature.

First author, year (reference)	Number of patients	Type of study	Frequency intracerebral hemorrhage (%)	Very old patients (%) (mean age)*	Clinical features (percentage of patients)	In-hospital mortality (%)
Gates, 1986 [37]	5	Clinical series	Not stated	None	Altered consciousness 100	20
Verma et al., 1987 [26]	12	Clinical series	Not stated	Not stated	Not stated	33.3
Chan and Mann, 1988 [38]	22	Clinical series	Not stated	Not stated	Not stated	23
Darby et al., 1988 [39]	7	Clinical series	3.1		Altered consciousness 100	28.5
Jayakumar et al., 1989 [40]	15	Clinical series	8.8	None	Altered consciousness 73	46
Ara et al., 1991 [11]	42	Clinical series	7.1	Not stated	No	23.8
Angelopoulos et al., 1995 [24]	14	Clinical series	Not stated	Not stated	Headache 78 Altered consciousness 71 Nausea/vomiting 71	36
Rohde et al., 1995 [33]	20	Clinical series	Not stated	Not stated	Not stated	5%
Tembl et al., 1997 [27]	8	Clinical series	Not stated	Not stated	Not stated	0%
Martí-Fàbregas et al., 1999 [13]	13	Clinical series	Not stated	7.7% (60 years)	Headache 69 Altered consciousness 69	23
Passero et al., 2002 [25]	26	Clinical series	Not stated	Not stated	Headache 60 Altered consciousness 44	42
Hameed et al., 2005 [10]	15	Clinical series	2	Not stated (56 years)	Not stated	13.3
Flint et al., 2008 [12]	15	Clinical series	2.7	Not stated (64 years)	Headache 67 Nausea/vomiting 47 Altered consciousness 80	7
Giray et al., 2009 [41]	24	Clinical series	Not stated	0	Headache 100 Altered consciousness 83 Nausea/vomiting 41.5	42
Present series, 2012	12	Hospital-based stroke registry	3.3	41.7% (78.9 years)	Headache 50 Altered consciousness 66.7	41.7

*Age 85 years or more.
